# Draft Genome Sequence of Bacillus thuringiensis ZZQ-130 with Multiple Pesticidal Genes, Isolated from Caka Salt Lake, China

**DOI:** 10.1128/mra.00887-21

**Published:** 2022-02-10

**Authors:** Ziqiang Zheng, Xiaojie Lan, Qi Wang, Chengjun Zhang, Guoqiang Huang, Jianping Zhang, Hongxun Wang

**Affiliations:** a Xinjiang Production & Construction Crops Key Laboratory of Protection and Utilization of Biological Resources in Tarim Basin, Tarim University, Xinjiang, China; b College of Life Science and Technology, Wuhan Polytechnic University, Wuhan, China; c Division of Animal Infectious Disease, Huazhong Agricultural University, Wuhan, Hubei, China; d College of Life Science and Technology, Huazhong Agricultural University, Wuhan, China; Indiana University, Bloomington

## Abstract

Bacillus thuringiensis is a typical pesticide, with global application for over 40 years. Here, we report the draft genome sequence of B. thuringiensis ZZQ-130 from a salt lake; this strain has 31 pesticidal genes, including five *cry* genes, one *vip* gene, two *vpa* genes, and two *vpb* genes.

## ANNOUNCEMENT

Tribolium castaneum is a stored grain pest which is responsible for global grain losses (1). Biopesticides are gradually being used as alternatives to chemical pesticides. Bacillus thuringiensis (Bt) is the most useful bacterial pesticide, with plentiful pesticidal proteins such as crystal proteins (Cry), cytolytic proteins (Cyt), and vegetative insecticidal proteins (Vip) (2). Identification of novel pesticidal genes is always the main work of fundamental research and production development involving Bt pesticides. Hostile ecological environments usually contain Bt strains with novel pesticidal genes. ZZQ-130 was isolated using the classical spread plate method from sediment soil from Caka Salt Lake, Qinghai Province, China, and cultured in salty (6%) LB medium at 28°C. ZZQ-130 was then identified as a Bt strain using the 16S rRNA gene with an identity of 100% to B. thuringiensis FDAARGOS_796 (coverage, 100%).

Genomic DNA from ZZQ-130 at the logarithmic growth phase in liquid salty (6%) LB medium was isolated as per the previously modified alkaline lysis method of Andrup et al. (3). A DNA library was prepared using the NEXTflex rapid DNA-Seq kit, after fragmentation using a Covaris M220 ultrasonicator, and sequenced using an Illumina HiSeq X Ten sequencer. A total of 3,807,467 paired-end reads of 150 bp were obtained, with a sequencing depth over 170-fold. A quality check of the Illumina reads was performed using fastp v0.20.0 (4); then, the reads were assembled using the “Assemble” module of the PGCGAP pipeline (5). The command line and parameters are “pgcgap --Assemble --platform illumina --assembler auto --filter_length 200 --ReadsPath Reads/Illumina --reads1 _1.fastq.gz --reads2 _2.fastq.gz --kmmer 81 --threads 4 --suffix_len 11.” The filtered contigs over 200 bp were annotated using the NCBI Prokaryotic Genome Annotation Pipeline (PGAP) (6).

The draft genome of strain ZZQ-130 has a size of 6,677,513 bp, derived from 351 contigs, with an *N*_50_ value of 119,425 bp and a GC content of 34.72%. Whole-genome alignment and assembly improvement were conducted using RagTag (7), with B. thuringiensis YBT-1520 (8) as the reference genome. The command line and parameters are “ragtag.py scaffold -f 1200 -t 10 ref/YBT-1520.fasta output_dir/ZZQ-130.fasta.” The scaffolded genome of ZZQ-130 harbors a chromosome with a size of 5,383,145 bp, and eight plasmids, including pB130-1 (305,619 bp), pB130-2 (42,407 bp), pB130-3 (39,632 bp), pB130-4 (32,648 bp), pB130-5 (28,563 bp), pB130-6 (14,861 bp), pB130-7 (8,352 bp), and pB130-8 (8,091 bp).

To analyze the pesticidal factors, a whole genomic search of bacterial pesticidal proteins was performed using BLASTP within the online pipeline BtToxin_Digger (https://bcam.hzau.edu.cn/BtToxin_Digger/index.php) (9). Thirty-one pesticidal proteins, including five rank 4 (identity, >95%) *cry* genes (K7H03_22040, K7H03_22045, K7H03_22070, K7H03_22070, and K7H03_27130), one rank 4 *vip* gene (K7H03_22005), one rank 1 (identity, <45%) *vpa* (*V*i*p*2—the *a*ctive component of the Vpa/Vpb binary toxin) gene (K7H03_29570), one rank 2 (identity, 45% to 76%) *vpa* gene (K7H03_27065), and two rank 2 *vpb* (*V*i*p*1—the binding component of the Vpa/Vpb binary toxin) genes (K7H03_29535 and K7H03_29120), were identified. All the rank 4 *cry* and *vip* genes may be responsible for the pesticidal activity, although their novelty has decreased. The genes above ranks 1 and 2 may encode novel pesticidal proteins and mechanisms and need further research.

### Data availability.

The whole-genome shotgun project of strain ZZQ-130 has been submitted to GenBank under accession number JAIOAD000000000 and assembly accession number GCA_020005185.1. The raw draft genome data have been deposited in the NCBI Sequence Read Archive under accession number SRR15827360, BioSample accession number SAMN21033747, and BioProject accession number PRJNA758751.
